# A Fully Integrated Monolithic Monitor for Aging-Induced Leakage Current Characterization †

**DOI:** 10.3390/s26010064

**Published:** 2025-12-22

**Authors:** Emmanuel Nti Darko, Saeid Karimpour, Daniel Adjei, Kelvin Tamakloe, Degang Chen

**Affiliations:** Department of Electrical and Computer Engineering, Iowa State University, Ames, IA 50011, USA

**Keywords:** ADC, leakage current monitor, TDDB, reliability, digitizer, SAR

## Abstract

This paper presents a precision, wide-dynamic-range leakage current sensor tailored for in-situ monitoring of aging mechanisms such as Time-Dependent Dielectric Breakdown (TDDB) in both active and passive components. The proposed architecture supports high-voltage stress and is fully monolithic, integrating a current-to-voltage front-end, tunable-gain amplifier, and a successive approximation register (SAR) analog-to-digital converter (ADC). To validate the concept, a discrete-component prototype was implemented and evaluated across a leakage current range of 1 nA to 1 μA. The sensor achieves 12-bit resolution with measured integral non-linearity (INL) and differential non-linearity (DNL) within ±1.5 LSB and ±0.3 LSB, respectively. Compared to prior monitors, the design enables linear current digitization and supports high-voltage stress, features essential for accurate and scalable TDDB characterization. Applications include embedded reliability monitoring in power converters, analog building blocks, and large-scale aging test arrays.

## 1. Introduction

Modern integrated circuits are increasingly deployed in mission-critical applications such as automotive and biomedical systems [1,2,3], where long-term reliability is a fundamental requirement. These circuits are routinely exposed to harsh environmental and electrical stress that can lead to progressive degradation or catastrophic failure. To mitigate these risks, embedded sensing features are becoming integral to System-on-Chip (SoC) design, enabling real-time health monitoring of on-chip devices and interconnects.

A key reliability challenge in scaled technologies stems from the fact that although transistors and interconnects continue to shrink, supply voltages have not scaled proportionally due to limitations in noise margins and I/O compatibility [4]. This mismatch results in elevated electric fields across thin gate dielectrics and densely packed metal lines, accelerating degradation mechanisms such as Time-Dependent Dielectric Breakdown (TDDB) and Stress-Induced Leakage Current (SILC). While commercial source-measure units (SMUs) are commonly used for leakage current measurement, their large form factor, high cost, and limited scalability make them unsuitable for on-chip or field-deployable monitoring. Furthermore, their complexity and power requirements increase significantly when broad dynamic range support is needed [5].

Among the most prominent degradation phenomena are Time-Dependent Dielectric Breakdown (TDDB), which is driven by progressive defect formation and charge transport through insulating materials under sustained electric stress [6,7]. These mechanisms are particularly concerning in modern systems-on-chip (SoCs), where dense interconnects, stacked dielectrics, and high-performance operation converge to create highly stress.

While much of the existing reliability literature focuses on active devices such as MOSFET gate oxides, passive structures—including metal–insulator–metal (MIM) and vertical natural capacitors (VNcaps)—are increasingly vulnerable under similar stress conditions. VNcaps, in particular, have become widely adopted in advanced CMOS processes due to their superior capacitance density, layout efficiency, and low fabrication cost [8,9,10,11]. These structures rely on multilayered metal–dielectric stacks formed through the backend-of-line (BEOL) process and are increasingly utilized in analog and mixed-signal domains, where large-area capacitors are essential for signal conditioning, filtering, and biasing. However, the same physical properties that make VNcaps attractive also expose them to reliability risks: their vertically stacked geometry, coupled with thin dielectrics and dense via interconnects, results in locally intensified electric fields that accelerate dielectric aging and TDDB onset.

Despite their pervasive use, the reliability of VNcaps remains comparatively understudied. Existing on-chip sensing approaches have largely targeted gate leakage monitoring in transistors, often assuming low-voltage operation and limited dynamic range. These designs are typically unsuitable for passive components, particularly under the elevated terminal voltages required to induce accelerated stress for TDDB characterization. Furthermore, commercial instruments such as source-measure units (SMUs), though highly accurate, are fundamentally unsuitable for embedded or large-scale deployment due to their high cost, bulky size, and limited scalability for multi-site or in-field monitoring [5,12].

Several prior works have proposed on-chip sensors for monitoring gate leakage in MOS transistors under TDDB stress [13,14,15,16,17]. While these designs demonstrate effective detection of early oxide degradation in active devices, they are generally optimized for low-voltage conditions and lack support for passive components such as VNcaps. Notably, most do not accommodate the high terminal voltages required for accelerated breakdown testing in large-area capacitors, nor do they integrate wide dynamic range digitization necessary for sub-nanoampere current resolution. These limitations motivate the need for a more robust and versatile sensing solution.

This paper introduces a monolithic, low-leakage current sensing architecture capable of operating over a wide dynamic range. The proposed design is tailored to withstand high-voltage stress conditions, making it well-suited for the in situ monitoring and characterization of TDDB in both high-power MOSFETs and large-area passive capacitors. The sensor functions by converting leakage current into a corresponding voltage using a precision current-to-voltage front-end, which is subsequently digitized using an integrated, on-chip successive approximation register (SAR) analog-to-digital converter. This fully embedded solution removes the dependency on bulky and expensive external ADCs or SMUs that are commonly used in traditional reliability measurement systems.

Additionally, a tunable gain stage is incorporated into the architecture to dynamically adjust the measurement range based on the expected leakage levels, thereby improving both sensitivity and resolution across a broad spectrum of current magnitudes and stress conditions. By eliminating external instrumentation and supporting high-voltage operation, the proposed sensor architecture offers a scalable and cost-effective solution for accelerated TDDB studies, long-term degradation tracking, and real-time reliability monitoring in safety-critical applications such as automotive, biomedical, and aerospace electronics.

The rest of this manuscript is structured as follows. Section 2 details the proposed sensor architecture and provides a comprehensive and rigorous analytical derivation of its operation. Section 3 discusses the detailed implementation of the proposed sensor. Section 4 presents experimental results. Section 5 offers a comparative evaluation with prior work, emphasizing the benefits, limitations, and potential applications of the proposed solution. Finally, Section 6 concludes the paper by summarizing the main contributions and insights.

## 2. Proposed Leakage Current Monitor

Accurate characterization of ultra-low leakage currents—particularly those associated with reliability degradation mechanisms such as TDDB demands a sensing system capable of operating over an exceptionally wide dynamic range. This is critical for observing leakage behavior across different stages of device aging, from initial trap-assisted tunneling currents in early stress phases to pronounced leakage near breakdown, where current magnitudes may vary by several orders. To address this challenge, the proposed sensor architecture is engineered to support a broad input current range without compromising resolution or linearity.

**Figure 1:**
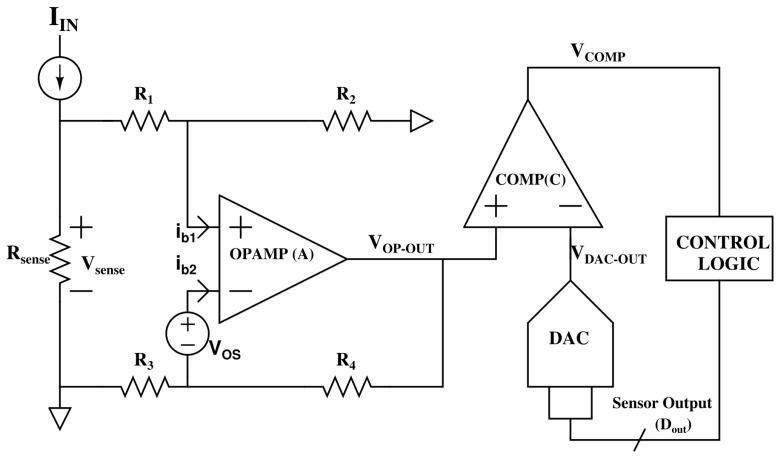
Proposed circuit for low leakage current monitoring.

A key feature of the design is its programmable gain adjustment mechanism, which allows flexible scaling of the input-to-output transfer characteristic. This enables the system to adapt dynamically to varying leakage levels, without modifying the core sensing or digitization path. Such tunability is essential for supporting both accelerated stress testing in controlled environments and in situ monitoring in field-deployed systems. The complete architecture—including the precision current-to-voltage front-end, variable-gain amplifier (VGA), on-chip SAR ADC, and digital control logic—is illustrated in Figure 1, and forms the foundation of a compact, fully integrated TDDB characterization platform.

The leakage current of interest, denoted as $I_{IN}$, is routed through a sensing resistor $R_{\mathrm{sense}}$, generating a voltage drop $V_{\mathrm{sense}}=I_{IN}\cdot R_{\mathrm{sense}}$ across its terminals. In the context of TDDB characterization, this input current originates from a stressed device under test (DUT)—typically a high-voltage MOSFET or a VNcap—subjected to accelerated electrical stress. As the DUT ages, its dielectric integrity gradually degrades, leading to an exponential increase in leakage current. Consequently, the sensed voltage $V_{\mathrm{sense}}$ serves as a direct indicator for monitoring the degradation trajectory and reliability status of the device [18].

To amplify this low signal, $V_{\mathrm{sense}}$ is applied to the non-inverting input of a precision, high-gain operational amplifier *A*, which is configured with a four-resistor feedback network consisting of $R_{1}$ through $R_{4}$. This network establishes a programmable closed-loop gain that enables small leakage-induced voltages to be upscaled into a suitable range for digitization. However, due to the resistive nature of the feedback path, the input current $I_{IN}$ undergoes partial division between the sensing resistor $R_{\mathrm{sense}}$ and the parallel feedback path via $R_{1}$. This current splitting must be carefully modeled and compensated for to ensure accurate reconstruction of the original leakage current, especially under low-current, high-gain operating conditions.

Applying Kirchhoff’s Current Law (KCL) at the $V_{\mathrm{sense}}$ node yields:

(1) $$I_{IN}=\frac{V_{\mathrm{sense}}}{R_{\mathrm{sense}}}+\frac{V_{\mathrm{sense}}-V_{in+}}{R_{1}}$$

This equation shows that the total sensed current $I_{IN}$ consists of two components: one flowing through the sensing resistor $R_{\mathrm{sense}}$, and the other through the feedback resistor $R_{1}$ into the amplifier’s feedback path. Since the operational amplifier has high gain and very low input bias current, we assume negligible current enters the amplifier input itself. Rearranging Equation (1), the voltage at the sensing node, $V_{\mathrm{sense}}$, can be expressed as:

(2) $$V_{\mathrm{sense}}=\frac{R_{\mathrm{sense}}\left(V_{in+}+I_{IN}R_{1}\right)}{R_{1}+R_{\mathrm{sense}}}$$

Applying Kirchhoff’s Current Law (KCL) at the inverting input node of the amplifier, denoted as $V_{{in}^{-}}$, we obtain:

(3) $$\frac{V_{os}-V_{in-}}{R_{3}}=\frac{V_{in-}-V_{os}-V_{\text{OP-OUT}}}{R_{4}}+i_{b2}$$

where $i_{b2}$ is the input bias current and $V_{os}$ is the input-referred offset voltage.

Solving for the output voltage $V_{\text{OP-OUT}}$ in terms of $V_{in-}$:

(4) $$V_{\text{OP-OUT}}=i_{b2}R_{4}+\left(V_{in-}-V_{os}\right)\left(1+\frac{R_{4}}{R_{3}}\right)$$

The voltage at the non-inverting input, $V_{in+}$, is derived from the sensing node $V_{\mathrm{sense}}$ through a resistive divider consisting of $R_{1}$ and $R_{2}$. Accounting for the input bias current $i_{b1}$, the voltage relationship is given by:

(5) $$\frac{V_{\mathrm{sense}}-V_{in+}}{R_{1}}=i_{b1}+\frac{V_{in+}}{R_{2}}$$

Rewriting Equation (5) gives:

(6) $$V_{\mathrm{sense}}=V_{in+}\left(\frac{R_{1}+R_{2}}{R_{2}}\right)+i_{b1}R_{1}$$

Now, by equating the two expressions for $V_{\mathrm{sense}}$ obtained in Equations (2) and (6), we can isolate and derive an explicit expression for the amplifier’s non-inverting input voltage, $V_{in+}$:

(7) $$V_{in+}=\frac{I_{IN}\cdot R_{1}R_{2}R_{\mathrm{sense}}-i_{b1}\cdot R_{1}R_{2}\left(R_{1}+R_{\mathrm{sense}}\right)}{\left(R_{1}+R_{2}\right)\left(R_{1}+R_{\mathrm{sense}}\right)-R_{2}R_{\mathrm{sense}}}$$

Also, the output voltage of an operational amplifier is given by:

(8) $$V_{\text{OP-OUT}}=A_{o}\left(V_{in+}-V_{in-}\right)$$

Solving for $V_{in+}$ by combining Equations (4) and (8), and eliminating the dependence on $V_{in-}$, we arrive at the following expression:

(9) $$V_{in+}=\frac{V_{\text{OP-OUT}}\left(R_{3}\left(A_{o}+1\right)+R_{4}\right)-A_{o}R_{3}R_{4}i_{b2}}{A_{o}\left(R_{3}+R_{4}\right)}+V_{os}$$

Equating Equations (7) and (9), and solving for $V_{\text{OP-OUT}}$, we derive an expression for the sensor’s output voltage as a function of the input leakage current and circuit parameters:

(10) $$\begin{array}{cc} V_{\text{OP-OUT}} & =\frac{1}{R_{3}\left(A_{o}+1\right)+R_{4}}\left[\frac{A_{o}\left(R_{3}+R_{4}\right)R_{1}R_{2}R_{\mathrm{sense}}}{\left(R_{1}+R_{2}\right)\left(R_{1}+R_{\mathrm{sense}}\right)-R_{2}R_{\mathrm{sense}}}\right]I_{IN}\\ \; & \;-\frac{i_{b1}}{R_{3}\left(A_{o}+1\right)+R_{4}}\left[\frac{R_{1}R_{2}\left(R_{1}+R_{\mathrm{sense}}\right)}{\left(R_{1}+R_{2}\right)\left(R_{1}+R_{\mathrm{sense}}\right)-R_{2}R_{\mathrm{sense}}}\right]\\ \; & \;+\frac{A_{o}R_{3}R_{4}i_{b2}-V_{os}A_{o}\left(R_{3}+R_{4}\right)}{R_{3}\left(A_{o}+1\right)+R_{4}} \end{array}$$

This equation shows the linear relationship between the output voltage $V_{\text{OP-OUT}}$ and the sensed current $I_{IN}$, where the first term represents the gain (or slope) of the sensor. The remaining terms contribute to the output offset, primarily arising from non-idealities such as finite amplifier gain and input bias currents. Under ideal conditions, where $A_{o}\gg 1$ and the bias currents are negligible, the expression simplifies significantly to:

(11) $$V_{\text{OP-OUT}}=I_{IN}\cdot \frac{A_{o}\left(R_{3}+R_{4}\right)R_{1}R_{2}R_{\mathrm{sense}}}{\left(R_{3}A_{o}+R_{4}\right)\left[\left(R_{1}+R_{2}\right)\left(R_{1}+R_{\mathrm{sense}}\right)-R_{2}R_{\mathrm{sense}}\right]}$$

To ensure symmetry and resistor matching, we choose $R_{1}=R_{3}$ and $R_{2}=R_{4}$. With this configuration, the gain term simplifies, yielding the slope of the transfer characteristic as:

(12) $$s=\frac{R_{2}R_{\mathrm{sense}}\left(R_{1}+R_{2}\right)}{R_{1}\left(R_{1}+R_{2}+R_{\mathrm{sense}}\right)}$$

This voltage is fed to the comparator *C*, which compares it against a reference voltage $V_{\mathrm{COMP}}$. The comparator output controls the successive approximation register (SAR) logic, which iteratively generates the digital output code $D_{\mathrm{OUT}}$. This digital code is then applied to a DAC whose output updates $V_{\mathrm{comp}}$, forming a closed-loop system that converges to the digital representation of the input voltage.

At the end of the SAR search process, the comparator output settles, and the final DAC voltage converges to the amplified output of the sensing path. This equilibrium condition is expressed as:

(13) $$V_{\text{OP-OUT}}=V_{\mathrm{COMP}}$$

The DAC output voltage, $V_{\mathrm{COMP}}$, is determined by the reference voltage $V_{\mathrm{REF}}$ and the digital output code $D_{\mathrm{OUT}}$, such that:

(14) $$V_{\mathrm{COMP}}=V_{\mathrm{REF}}\cdot D_{\mathrm{OUT}}$$

Substituting Equation (14) into Equation (13) yields the final expression that links the amplified sensor output to the SAR digital result:

(15) $$V_{\text{OP-OUT}}=V_{\mathrm{REF}}\cdot D_{\mathrm{OUT}}$$

Thus, substituting the slope expression into this relation gives the final transfer function of the sensor in terms of system-level parameters:

(16) $$s_{A}=\frac{1}{V_{\mathrm{REF}}}\cdot \left[\frac{R_{2}R_{\mathrm{sense}}\left(R_{1}+R_{2}\right)}{R_{1}\left(R_{1}+R_{2}+R_{\mathrm{sense}}\right)}\right]$$

This expression highlights that the sensor’s gain—or slope of the transfer function—is entirely determined by the passive components in the feedback and sensing paths. As such, it can be easily adjusted through resistor selection, providing a straightforward means of tuning both resolution and dynamic range. Furthermore, since the offset term in Equation (10) does not depend on the input current $I_{IN}$, it can be effectively compensated through digital calibration or post-processing, enhancing the overall robustness of the system against analog nonidealities.

## 3. Board Level Implementation

**Figure 2:**
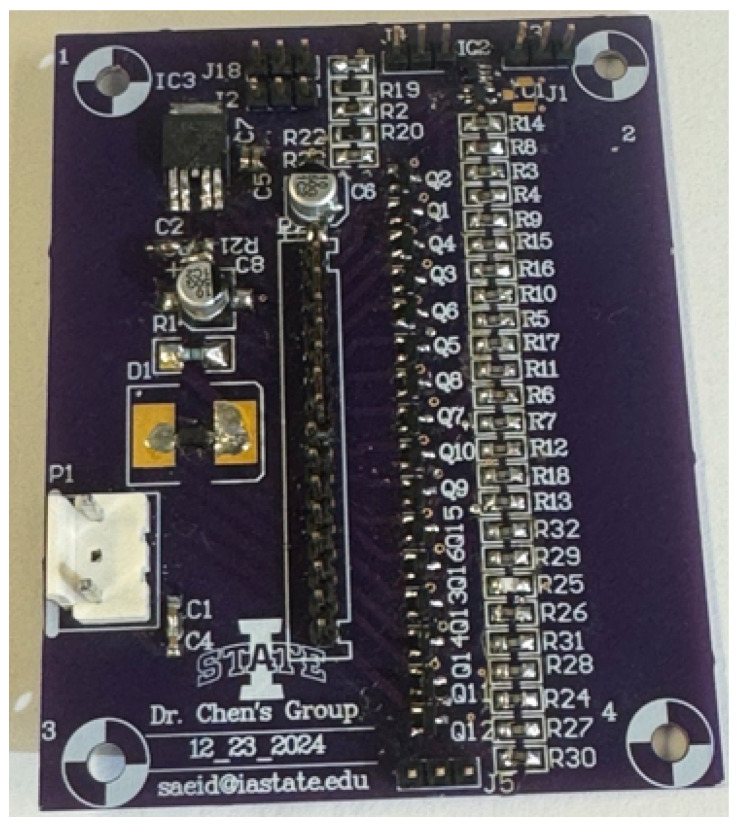
Fabricated PCB Prototype of Proposed circuit.

To experimentally validate the proposed leakage current sensing architecture, a board-level prototype was designed and fabricated using discrete off-the-shelf components, as shown in Figure 2. This prototype enables practical verification of the sensor’s functionality across a wide dynamic range and under diverse biasing conditions that emulate real-world TDDB stress scenarios. By evaluating performance in this controlled yet flexible environment, we can assess key metrics such as linearity, sensitivity, dynamic range, and temperature stability prior to full chip-level integration.

Precision surface-mount resistors were selected to form the sensing and gain network, ensuring minimal drift and mismatch over temperature. Specifically, the sensing resistor was chosen as $R_{\mathrm{sense}}=100\;k\Omega$, while the gain resistors were configured as $R_{1}=R_{3}=10\;k\Omega$ and $R_{2}=R_{4}=1\;M\Omega$, yielding a nominal closed-loop gain of 100. This gain was determined based on target leakage current levels ranging from sub-nanoampere to several microamperes, ensuring sufficient resolution and swing at the amplifier output. All resistors used had temperature coefficients of less than 25 ppm/°C to minimize thermal variation.

To further enhance calibration flexibility and account for board-level parasitic effects or resistor tolerance errors, a high-precision potentiometer was incorporated into the feedback path. This adjustable element allows fine-tuning of the gain after assembly, ensuring accurate mapping of input current to output voltage across the full dynamic range. This is particularly important for applications involving accelerated stress testing, where precise current measurements are essential to extract meaningful reliability metrics.

The core amplifier used in the design is the Texas Instruments **OPA392**, a low-power, rail-to-rail input/output operational amplifier featuring low input offset voltage (<1 mV) and high common-mode rejection, making it well-suited for low-current precision measurements [19]. One OPA392 serves as the transimpedance amplifier in the current-to-voltage front-end, while a second OPA392 is reconfigured in comparator mode to perform analog comparison within the successive approximation register (SAR) ADC loop. Although the OPA392 is not optimized as a high-speed comparator, it provides sufficiently sharp transitions for functional validation in this context.

The DAC used in the SAR loop is the **MCP4822**, a dual-channel, 12-bit voltage-output digital-to-analog converter from Microchip [20]. This DAC provides fine resolution and stable voltage steps for the SAR comparator, allowing accurate digital-to-analog conversion during the successive approximation process.

This setup ensures that the entire sensing current $I_{IN}$ flows through $R_{\mathrm{sense}}$, generating a well-defined voltage $V_{\mathrm{sense}}$, which is amplified and digitized by the embedded SAR logic. The use of discrete high-precision components provides confidence in the analog performance and allows for easy iteration during prototyping. The board-level prototype thus serves as a practical proof-of-concept for the sensor’s core architecture, demonstrating its capability to detect ultra-low leakage currents with high resolution, stability, and compatibility with standard CMOS integration in future on-chip implementations.

## 4. Experimental Results

This section presents the experimental validation of the proposed leakage current sensing architecture using the fabricated PCB prototype.

### 4.1. Measurement Setup and Control Flow

**Figure 3:**
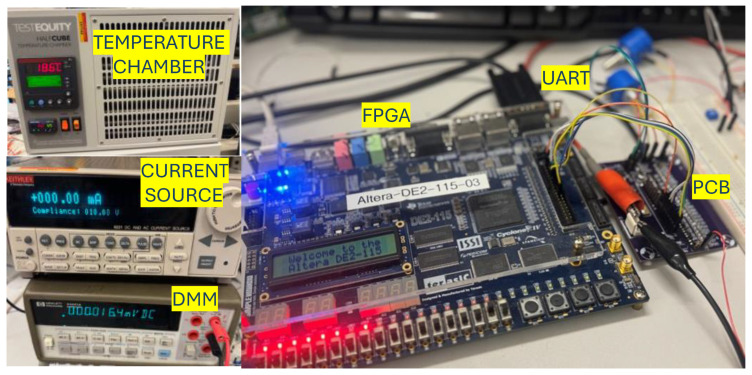
Measurement Setup for validating proposed circuit.

**Figure 4:**
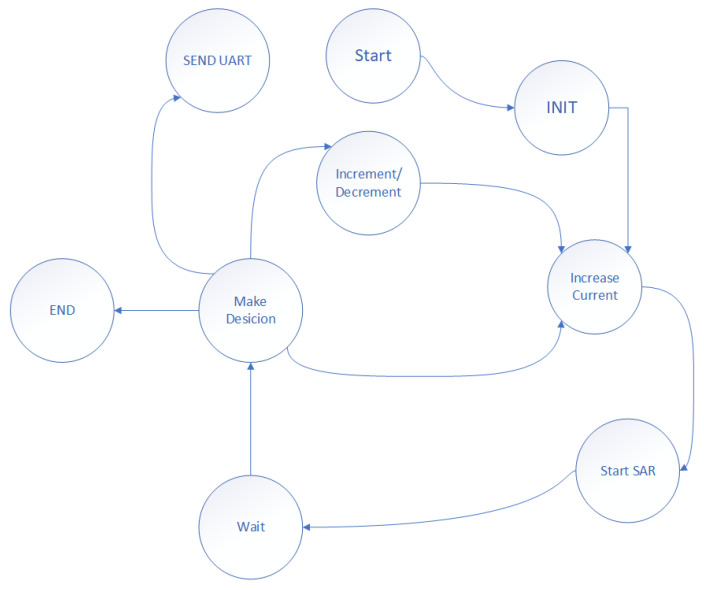
Finite State Machine Implemented on FPGA: State Transition Diagram.

As previously described, the sensor was implemented with discrete components to evaluate its functionality over a wide dynamic range. The measurement setup is shown in Figure 3. For this validation, the input current $I_{IN}$ is generated using an external precision current source and linearly swept across the target range. An FPGA governs the measurement process, initiating conversions and recording the corresponding sensor output code. The system operation is orchestrated by a finite state machine (FSM) implemented on the FPGA, as depicted in Figure 4.

The FSM begins in the **Start** state and transitions to an **INIT** state, where all internal variables and control signals are initialized. It then proceeds to the **Increase Current** state, incrementally stepping the input current through a predefined set of values. Following this, the FSM transitions to the **Start SAR** state, where it initiates a SAR conversion to digitize the sensor’s analog output.

During the SAR process, the system moves into the **Make Decision** state to evaluate the comparator output and determine whether the DAC code should be incremented or decremented. Based on this evaluation, the FSM enters the **Increment/Decrement** state to update the DAC input accordingly. This process repeats in a closed-loop fashion until convergence.

Once the SAR search converges for a given input current, the FSM transitions to the **Send UART** state, where the final digital output code is transmitted via UART to a PC for logging and analysis. After a brief **Wait** state to ensure timing and stability, the FSM checks whether additional current steps remain. If so, the cycle repeats; otherwise, the FSM enters the **END** state to terminate the measurement sequence. This automated FSM-based loop ensures consistent and high-resolution tracking of the sensor’s response across the full leakage current range. The measurement process is repeatable, efficient, and scalable, enabling robust performance evaluation of the sensor under diverse biasing and stress conditions.

### 4.2. Measured Results

**Figure 5:**
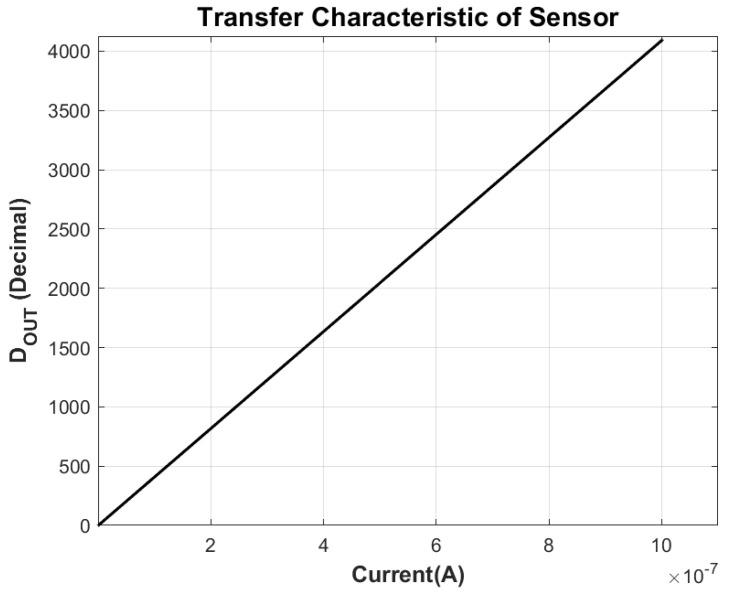
Transfer Characteristic of the Proposed Sensor at Nominal Conditions.

**Figure 6:**
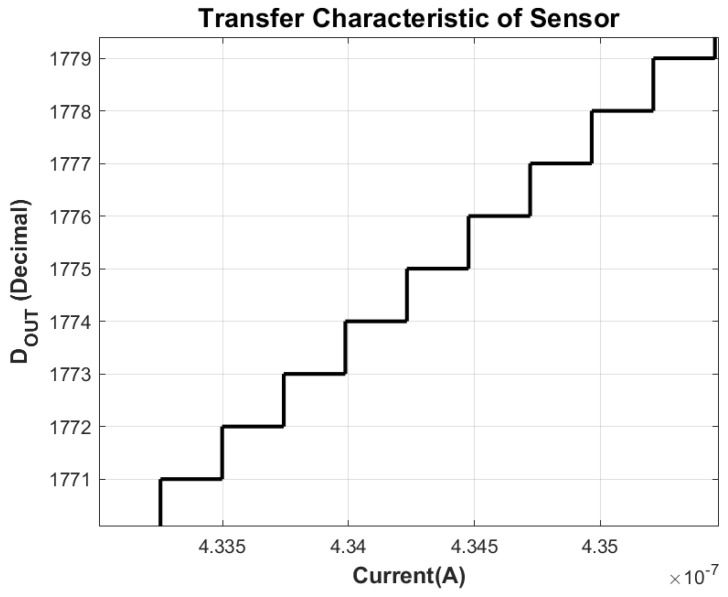
Zoomed Transfer Characteristic at Nominal Conditions.

The measured transfer characteristic of the proposed sensor is presented in Figure 5. A zoomed-in view is provided in Figure 6, clearly showing transitions of code with increasing current. The output codes exhibit a monotonic and highly linear response with respect to the applied input current, confirming the accuracy of the current-to-voltage conversion and embedded SAR digitization process.

**Figure 7:**
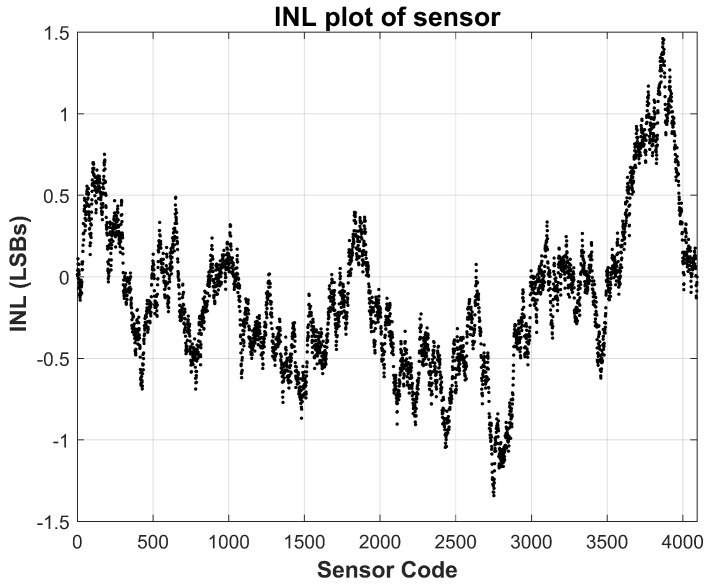
Measured Sensor Integral Non-Linearity (INL) at nominal 25 °C temperature. INL stays within ±1.5 LSBs.

**Figure 8:**
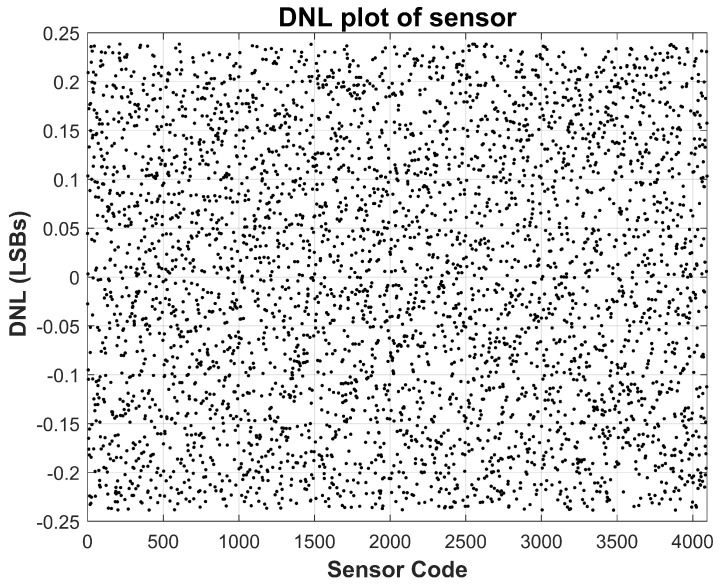
Measured Sensor Differential Non-Linearity (DNL) at nominal 25 °C temperature. DNL stays within ±0.25 LSBs.

The linearity of the sensor is evaluated using standard static performance metrics, namely integral non-linearity (INL) and differential non-linearity (DNL), based on measurements acquired at room temperature (approximately 25 °C). These metrics are calculated by comparing the measured transfer function against an ideal straight-line fit spanning the full-scale current range. As depicted in Figure 7 and Figure 8, the measured INL remains within ±1.5 LSBs across the entire operating range, while the DNL stays confined within ±0.3 LSBs. These values are well within acceptable limits for most analog-to-digital converter systems, confirming that the proposed sensor achieves excellent monotonicity and static linearity. Such performance is critical for applications where precise current tracking is necessary, including TDDB degradation monitoring and stress-acceleration characterization.

To further assess the robustness and environmental stability of the sensing architecture, the sensor is also characterized across a wide temperature range. Measurements are conducted at two extreme temperature corners: a cold corner of −40 °C and a hot corner of 125 °C. These test points were selected in accordance with widely adopted reliability qualification standards, such as AEC-Q100, which specify such extremes for automotive-grade device evaluation.

**Figure 9:**
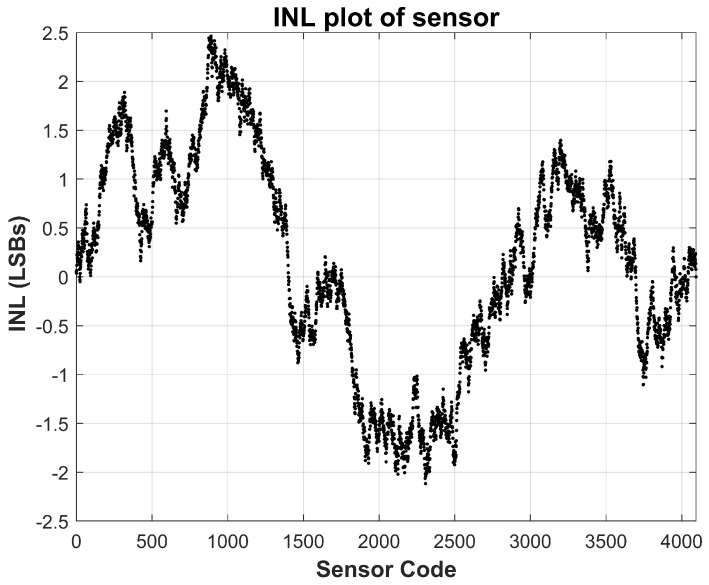
Measured Sensor Integral Non-Linearity (INL) at 125 °C temperature. INL stays within ±2.5 LSBs.

**Figure 10:**
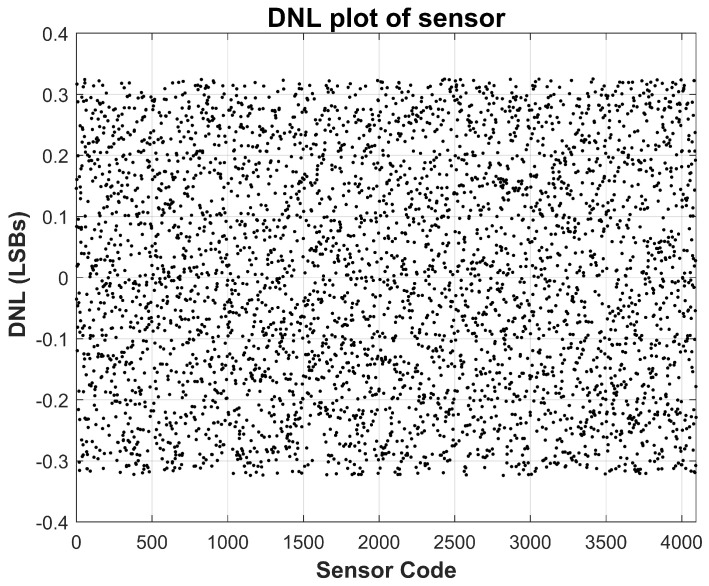
Measured Sensor Differential Non-Linearity (DNL) at 125 °C temperature. DNL stays within ±0.35 LSBs.

**Figure 11:**
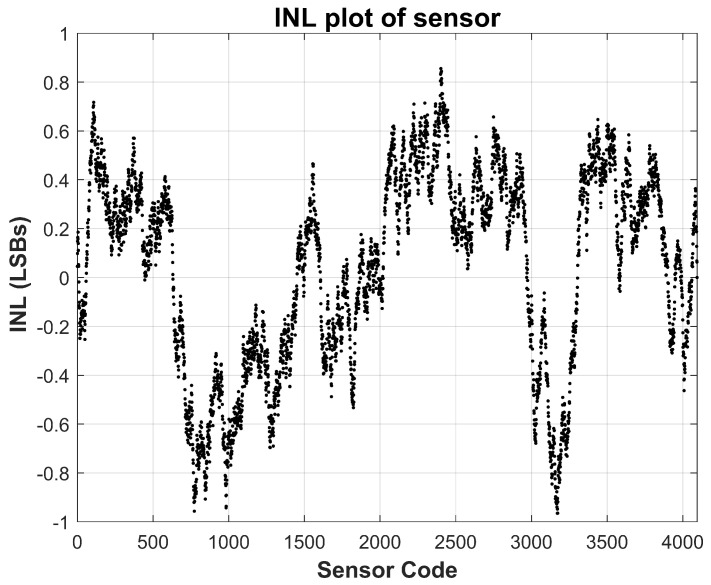
Measured Sensor Integral Non-Linearity (INL) at −40 °C temperature. INL stays within ±1 LSBs.

**Figure 12:**
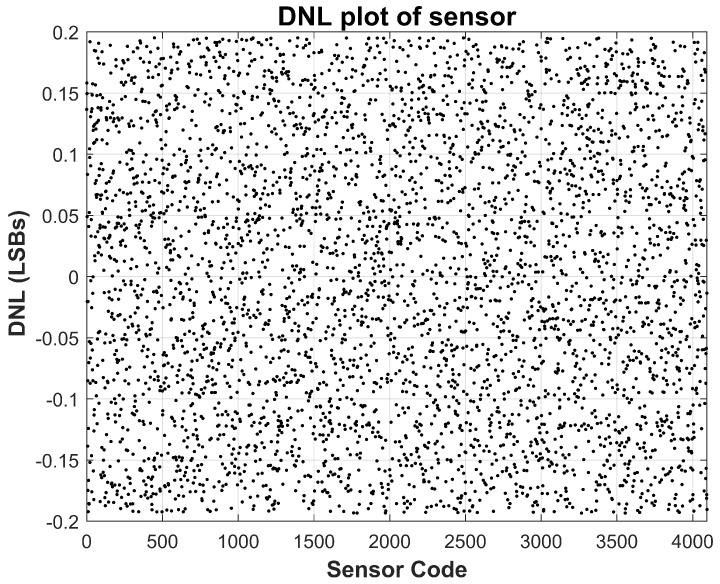
Measured Sensor Differential Non-Linearity (DNL) at −40 °C temperature. DNL stays within ±0.2 LSBs.

The results of the cold-temperature test are shown in Figure 9 and Figure 10, while those of the high-temperature test are presented in Figure 11 and Figure 12. Across both corners, the sensor preserves consistent behavior with minimal deviation in both INL and DNL, attesting to the robustness of the analog front-end and its temperature-compensated passive components. Importantly, the amplifier’s low offset drift and the precision of the gain-setting resistors ensure that the sensing transfer function remains stable across environmental variations.

These results validate the sensor’s capability to deliver accurate, linear, and repeatable measurements under challenging operating conditions. This makes the architecture particularly well-suited for deployment in mission-critical applications such as automotive electronics, aerospace systems, and long-term reliability monitoring of analog/mixed-signal SoCs.

Despite the large temperature variations, the sensor maintains a linear transfer function, with INL and DNL deviations remaining within acceptable limits across the full operating range. These results highlight the temperature resilience of the design and validate its suitability for harsh environment applications such as automotive, aerospace, and industrial monitoring.

This robustness under temperature stress is critical for aging and reliability studies, where accurate current sensing over extended time periods and fluctuating environmental conditions is required. The sensor’s ability to operate reliably across temperature corners also makes it a strong candidate for on-chip monitoring in qualification-grade ICs.

**Figure 13:**
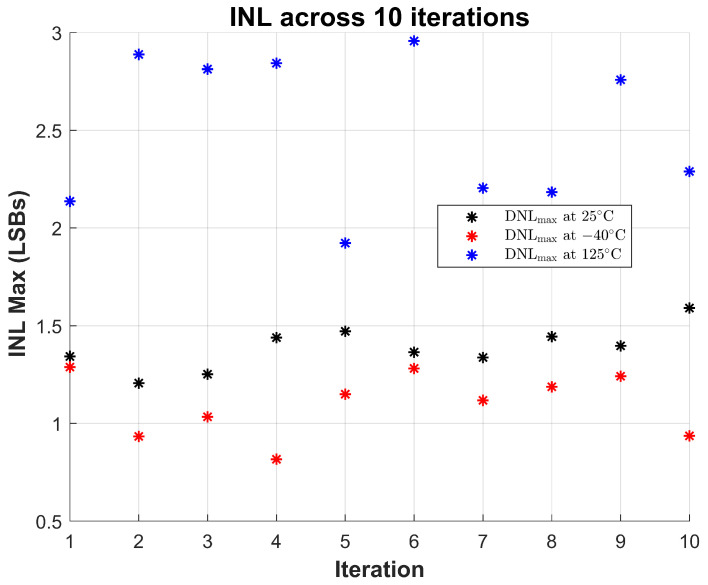
Maximum INL measured across 10 iterations at three temperature corners: room temperature (25 °C), cold (−40 °C), and hot (125 °C). The sensor exhibits consistent static linearity with ${\mathrm{INL}}_{\max}$ remaining below ±1.5 LSBs at all corners.

**Figure 14:**
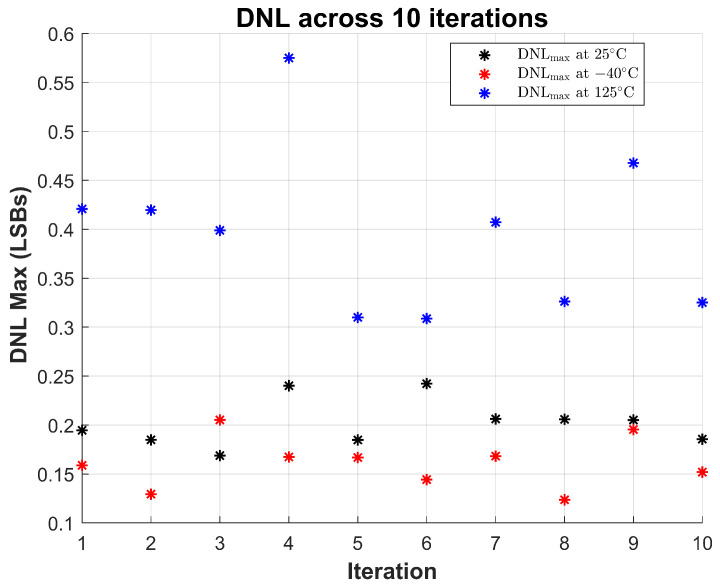
Maximum DNL measured across 10 iterations at three temperature corners: room temperature (25 °C), cold (−40 °C), and hot (125 °C). The sensor exhibits consistent static linearity with ${\mathrm{DNL}}_{\max}$ remaining below ±0.6 LSBs at all corners.

To validate the robustness of the proposed sensor, 10 repeated measurements were performed at room temperature (∼25 °C). The maximum observed INL and DNL across all trials are shown in Figure 13 and Figure 14, respectively. These results demonstrate the sensor’s stable linearity performance under consistent operating conditions, with INL values staying within ±1.6 LSBs and DNL within ±0.25 LSBs across all trials.

To further evaluate the resilience of the design under thermal stress, the same repeated measurements were performed at −40 °C and 125 °C. The results show that while the sensor exhibits increased variation at elevated temperatures—particularly in INL, which peaks around 2.9 LSBs at 125 °C—the overall behavior remains monotonic and within functional bounds. At cold temperatures (−40 °C), both INL and DNL values show slightly lower variation than at room temperature.

Notably, DNL remains well-controlled across all conditions, with maximum values below 0.6 LSBs even at the highest temperature. This indicates that code transitions remain uniform and glitch-free, a key requirement for reliable sensing. The results collectively demonstrate that the sensor maintains reliable linearity and code integrity under varying thermal conditions and across repeated trials, validating its suitability for long-term monitoring in reliability-critical applications.

## 5. Discussion

### 5.1. Key Contributions and Strengths

The proposed leakage current sensor introduces several important advancements over existing approaches. It achieves a wide dynamic range, accurately sensing currents from the sub-nanoampere to microampere level without the need for external amplifiers or gain switching networks. The design is also capable of operating under high terminal voltages, making it suitable for accelerated TDDB stress measurements in both active and passive components, such as MOSFETs and VNcaps.

Furthermore, the sensing architecture integrates a SAR ADC directly within the feedback loop, allowing for fully embedded digitization and eliminating the dependence on bulky external measurement instruments. The programmable resistive feedback network provides flexibility to tune the sensor’s gain and offset, simplifying calibration and enabling reuse across different measurement conditions and process corners.

The circuit is fully compatible with standard CMOS processes, which facilitates its integration into existing system-on-chip (SoC) platforms for long-term reliability and health monitoring. Finally, the complete functionality of the sensor has been validated at the board level using discrete components, demonstrating that precise, high-voltage, and low-leakage current sensing can be achieved with minimal cost and complexity. This combination of scalability, programmability, and measurement accuracy makes the proposed architecture a practical and versatile solution for embedded reliability evaluation.

### 5.2. Limitations

While the proposed sensor demonstrates strong performance in terms of resolution, dynamic range, and CMOS compatibility, several limitations remain. First, the current implementation is based on discrete components, which introduces parasitic effects and may not fully capture the behavior of an integrated on-chip realization.

Another important limitation is that the overall linearity of the system is strongly dependent on the performance of the DAC used in the SAR conversion process. The MCP4822, implemented as a resistor-string DAC, exhibits inherently superior DNL characteristics but limited INL linearity due to resistor gradient errors. Consequently, the measured DNL remains excellent, while the INL shows larger deviations. As with most ADC architectures, the linearity and resolution are ultimately constrained by the DAC’s intrinsic performance. A future implementation will address this by incorporating a higher-linearity DAC architecture or a digitally calibrated DAC to further enhance overall system linearity.

Additionally, temperature-dependent variations in the passive components and bias currents, though mitigated through careful selection of low-temperature-coefficient resistors, can still impact precision at extreme operating conditions. These effects, along with DAC-induced non-linearity, will be further optimized in a future fully integrated CMOS version of the sensor.

### 5.3. Comparison with Prior Work

Table 1 compares the proposed sensor with prior low-leakage current monitors. Unlike [13,14,15,16,17], which either lack high-voltage stress capability or use low-resolution logarithmic encoding schemes, the proposed design supports both linear current scaling and sustained high-voltage operation—key requirements for accurate TDDB stress evaluation in both active and passive devices. Moreover, this work demonstrates a 12-bit resolution across a wide input current range (1 nA–1 μA), significantly improving sensitivity and enabling fine-grained monitoring of early leakage signatures. The output format remains binary, which naturally aligns with SAR-based ADC systems and simplifies the overall control logic. While hybrid or thermometric encoding is certainly feasible in CMOS implementations, using a binary format keeps the architecture compact and low-power, reinforcing its suitability for on-chip integration and long-term in-field reliability monitoring in automotive and industrial environments.

### 5.4. Applications

The proposed sensor architecture is highly versatile and suitable for a range of reliability and aging-monitoring applications across both active and passive devices. Its ability to track ultra-low leakage currents under elevated terminal voltages makes it particularly well-suited for TDDB characterization of gate oxides and vertical natural capacitors (VNcaps).

**Figure 15:**
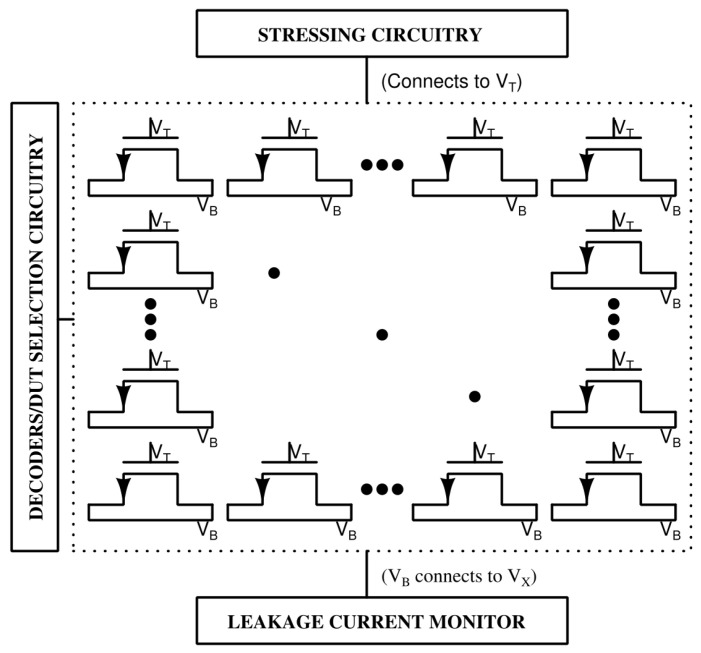
Application: Array of DUT Characterization.

As shown in Figure 15, the sensor can be integrated with large-area capacitor arrays or gate stacks to enable parallel stress testing, supporting statistical reliability analysis of multiple DUTs. The decoder-based selection scheme allows flexible routing of stress voltages and measurement channels, making the architecture scalable for large test arrays.

**Figure 16:**
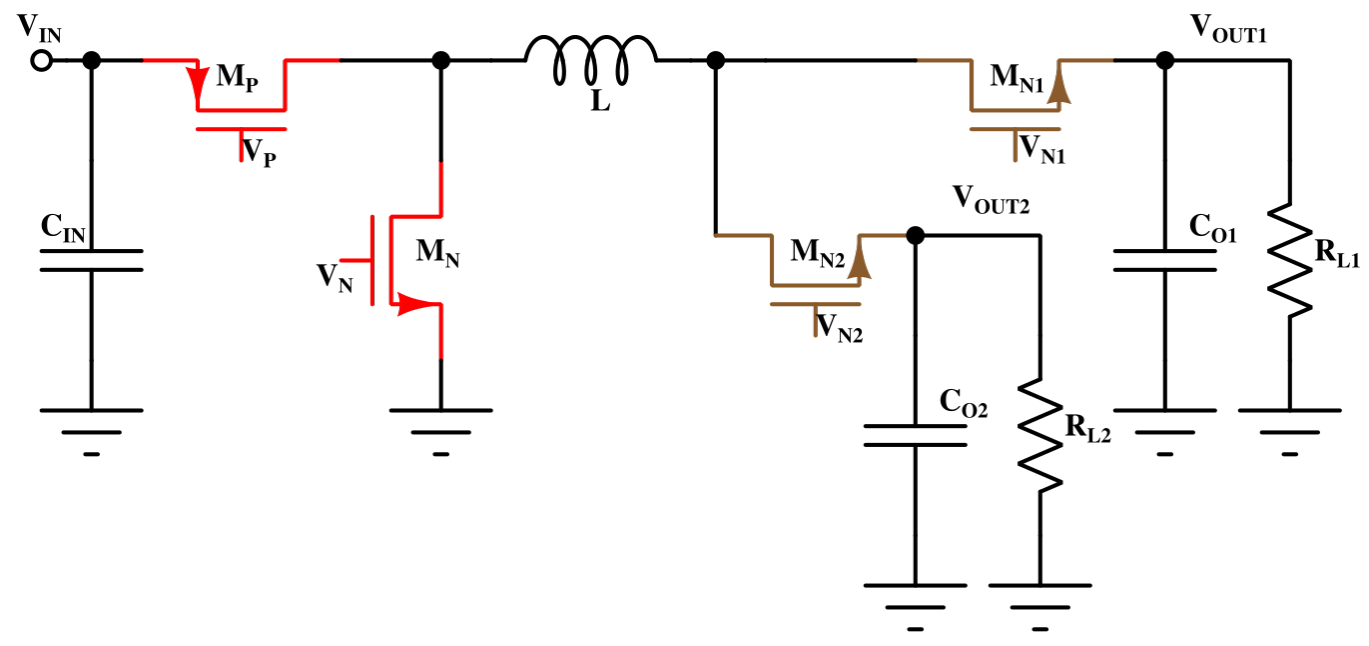
Application of the proposed sensor in a SIMO (Single-Input Multiple-Output) converter. Power MOSFETs in such converters are especially vulnerable to breakdown under high-voltage stress. A replica device monitored using the leakage sensor enables in situ TDDB tracking and predictive reliability assessment.

Beyond standalone reliability studies, the proposed sensor also supports in situ aging monitoring in critical analog/mixed-signal building blocks. One notable example is its application in DC-DC converter circuits, where long-term stability and reliability are essential. Figure 16 illustrates a Single Input Multiple Output (SIMO) converter architecture, in which multiple power transistors are driven from a shared input source. These power devices, especially the low-side switches, are often subjected to high electric fields and thermal stress, making them more susceptible to dielectric breakdown mechanisms. To monitor degradation in the field, a replica device can be incorporated into the power stage, biased similarly to the actual switches. The proposed leakage current sensor can then be connected to this replica to track stress-induced leakage over time. This approach enables early detection of reliability degradation, allowing for predictive maintenance or dynamic workload management in power delivery systems. Such integration is particularly relevant for automotive and industrial applications, where power converter failure can result in system-level faults or safety hazards.

## 6. Conclusions

This work presents a low-cost, high-resolution leakage current sensor capable of monitoring ultra-low currents across a wide dynamic range while supporting high-voltage stress conditions. The proposed architecture integrates a precision transimpedance amplifier, a tunable gain path, and a SAR ADC in a monolithic configuration, enabling accurate in situ tracking of TDDB-induced degradation. A board-level prototype built using discrete components validates the sensor’s linearity, resolution, and robustness across temperature, with measured INL and DNL within ±1.5 LSB and ±0.3 LSB, respectively, over a temperature range of −40 °C to 125 °C. Compared to prior monitors, this design uniquely supports linear current scaling, 12-bit resolution, and high-voltage stress compatibility, making it suitable for long-term reliability assessment in advanced CMOS technologies. Future work will explore fully integrated implementations and further improvements in linearity by adopting higher-performance DACs.

## Figures and Tables

**Table 1:** Comparison with Other Low Leakage Current Monitors.

Reference	Current Range (A)	Resolution (Bits)	Current Scale	HV Stress	Output Code Format
[13]	40 n–1.2 m	–	Linear	Yes	Binary
[14]	200 n–2 μ	–	Log	No	Binary
[15]	200 p–1 μ	4	Log	No	Therm+OneHot
[16]	1–200 μ	8	Linear	No	Binary
[17]	25 p–5 μ	6	Log	Yes	Therm+Binary
**This Work**	1 n–1 μ	12	Linear	Yes	Binary

## Data Availability

Most relevant information is provided in the article. Please contact the corresponding author for further enquiries or information including simulation data or/and codes.

## References

[1] (2014). AEC-Q100: Failure Mechanism Based Stress Test Qualification for Integrated Circuits, Automotive Electronics Council, Rev. H. https://studylib.net/doc/25765898/aec-q100-rev-h-base-document.

[2] Ferrati E. The reliability of the integrated circuits in automotive industry. Proceedings of the 1993 IEEE International Workshop on Defect and Fault Tolerance in VLSI Systems.

[3] Zhang Q., Li X., Zu T., Kang R. (2024). Belief reliability: A scientific exploration of reliability engineering. J. Syst. Eng. Electron..

[4] Cheng P., Mao L.F., Shen W.H., Yan Y.L. (2025). Electromigration failures in integrated circuits: A review of physics-based models and analytical methods. Electronics.

[5] Keysight Technologies (2024). Understanding the Importance of Leakage Current Measurement in Advanced Technology Nodes. Keysight Technologies, Tech. Overview 5990-9804. https://www.keysight.com/us/en/assets/7018-03320/technical-overviews/5990-9804.pdf.

[6] Darko E.N., Bhatheja K., Adjei D., Strong M., Chen D. On-chip monitoring of Time-Dependent Dielectric Breakdown (TDDB) using a novel leakage current sensor with digital output. Proceedings of the 2023 IEEE International Integrated Reliability Workshop (IIRW).

[7] Zhou H., Wang M., Bao R., Shen T., Wu E., Southwick R., Zhang J., Basker V., Guo D. TDDB reliability in gate-all-around nanosheet. Proceedings of the 2021 IEEE International Reliability Physics Symposium (IRPS).

[8] Lin C.Y., Avci U.E., Blount M.A., Grover R., Hicks J., Kasim R., Kundu A., Pelto C.M., Ryder C., Schmitz A. Reliability characteristics of a high density metal-insulator-metal capacitor on Intel’s 10+ process. Proceedings of the 2020 IEEE International Reliability Physics Symposium (IRPS).

[9] Hamada D.J.M., Roesch W.J. Reliability studies on thin metal-insulator-metal (MIM) capacitors. Proceedings of the ROCS Workshop—Reliability of Compound Semiconductors.

[10] Chen F., Ungar F., Fischer A.H., Gill J., Chinthakindi A., Goebel T., Shinosky M., Coolbaugh D., Ramachandran V., Siew Y.K. Reliability characterization of BEOL vertical natural capacitor using copper and low-k SiCOH dielectric for 65 nm RF and mixed-signal applications. Proceedings of the IEEE International Reliability Physics Symposium (IRPS).

[11] Fischer A.H., Lim Y.K., Riess P., Pompl T., Zhang B.C., Chua E.C., Keller W.W., Tan J.B., Klee V., Tan Y.C. TDDB robustness of highly dense 65 nm BEOL vertical natural capacitor with competitive area capacitance for RF and mixed-signal applications. Proceedings of the IEEE International Reliability Physics Symposium (IRPS).

[12] LaRow C., Chbili Z., Yap S.F., Kerber A., Nigam T. Fast TDDB monitoring for BEOL interconnect dielectrics. Proceedings of the IEEE International Integrated Reliability Workshop (IIRW).

[13] Keane J., Venkatraman S., Butzen P., Kim C.H. (2011). An Array-Based Test Circuit for Fully Automated Gate Dielectric Breakdown Characterization. IEEE Trans. Very Large Scale Integr. (VLSI) Syst..

[14] Nan H., Choi K. (2013). TDDB Monitoring and Compensation Circuit Design for Deeply Scaled CMOS Technology. IEEE Trans. Device Mater. Reliab..

[15] Bhatheja K., Jin X., Strong M., Chen D. (2021). Fast Gate Leakage Current Monitor with Large Dynamic Range. IEEE Trans. Circuits Syst. II: Express Briefs.

[16] Karimpour S., Sekyere M., Bruce I., Darko E.N., Chen D., McAndrew C.C., Garrity D., Jin X., Hatirnaz I., He C. (2024). Direct Current to Digital Converter (DIDC): A Current Sensor. Sensors.

[17] Darko E.N., Karimpour S., Adjei D., Johnson D., Bhatheja K., Chen D. (2025). Ultra-low leakage current sensor with large dynamic range. IEEE Trans. Circuits Syst. II: Express Briefs.

[18] Darko E.N., Karimpour S., Adjei D., Tamakloe K., Chen D. A simple monitor for tracking leakage current in capacitors for reliability assessment. Proceedings of the 2025 IEEE 34st Microelectronics Design & Test Symposium (MDTS).

[19] Texas Instruments (2022). OPA392: 36-V, Low Offset, Low-Drift, RRO, Precision Op Amp. Product Datasheet. https://www.ti.com/product/OPA392.

[20] Microchip Technology Inc. (2020). MCP4821/22: 12-Bit DAC with SPI Interface and Internal Reference, DS22244C. https://ww1.microchip.com/downloads/en/DeviceDoc/22244c.pdf.

